# Exogenous melatonin confers drought stress by promoting plant growth, photosynthetic capacity and antioxidant defense system of maize seedlings

**DOI:** 10.7717/peerj.7793

**Published:** 2019-10-11

**Authors:** Shakeel Ahmad, Muhammad Kamran, Ruixia Ding, Xiangping Meng, Haiqi Wang, Irshad Ahmad, Shah Fahad, Qingfang Han

**Affiliations:** 1 College of Agronomy/Key Laboratory of Crop Physio-ecology and Tillage in Northwestern Loess Plateau, Ministry of Agriculture, Northwest A&F University, Yangling, Shaanxi, China; 2 Key Laboratory of Agricultural Soil and Water Engineering in Arid and Semiarid Area, Ministry of Education/Institute of Water Saving Agriculture in Arid Areas of China, Northwest A&F University, Yangling, Shaanxi, China; 3 Institute of Agricultural Resources and Environment, Guangdong Academy of Agricultural Sciences, Guangzhou, Guangdong, China; 4 Department of Agriculture, University of Swabi, Swabi, Khyber Pakhtunkhwa, Pakistan

**Keywords:** Melatonin, Biomass, Chlorophyll, Antioxidants, Photosynthesis, Drought

## Abstract

Melatonin is an important biologically active hormone that plays a vital role in plant growth and development. In particular, it has been investigated for its roles in abiotic stress management. The current experiment was carried out to investigate the protective role of melatonin in photosynthetic traits and the antioxidant defense system of maize seedling under drought stress. Maize seedlings were subjected to drought stress (40–45% FC) after two weeks of seedling emergence, followed by a foliar spray (0, 25, 50, 75 and 100 µM) and soil drench of melatonin (0, 25, 50, 75 and 100 µM). Our results indicated that drought stress negatively affected maize seedling and decreased plant growth and development, biomass accumulation, reduced chlorophyll, and carotenoid content, and significantly declined photosynthetic rate and stomatal conductance. On the other hand, reactive oxygen species, soluble protein, and proline content increased under drought stress. However, the application of exogenous melatonin reduced the reactive oxygen species burst and enhanced the photosynthetic activity by protecting from damages through activation of various antioxidant enzymes under drought stress. Foliar application of 100 µM and soil drench of 50 µM melatonin was the most effective treatment concentrations under drought stress. Our current findings hereby confirmed the mitigating potential of melatonin application for drought stress by maintaining plant growth, improving the photosynthetic characteristics and activities of antioxidants enzymes.

## Introduction

Drought is a major environmental constraint which restricts plant growth and development (Cui et al., 2017; Naeem et al., 2018). It brings a wide range of morphological, physiological, and anatomical disruptions in plants (Liu et al., 2013; Cui et al., 2017). It also accelerates leaf senescence rate and degrades chlorophyll, which in turn decrease photosynthesis, reduce canopy size, resulting in lower crop yield and sometimes in total failure of the crop (Jiang et al., 2016; Kamran et al., 2018; Naeem et al., 2018). Drought stress induces the overproduction of reactive oxygen species (ROS) (Liang et al., 2019). Meanwhile, if these ROS are not eliminated, they cause oxidative damages to cell membranes, proteins, enzymes activity, RNA, and DNA (Dawood & El-Awadi, 2015; Cui et al., 2017).

Plants have evolved different strategies to counteract the harmful effects of drought stress and protect themselves against the detrimental effects of excessive ROS. The most relevant is the antioxidant defense system, comprised of enzymatic and non-enzymatic components that effectively scavenge the ROS and maintain a proper balance within the cells (Meloni et al., 2003). Enzymatic antioxidants include superoxide dismutase (SOD), ascorbate peroxidase (APX), peroxidase (POD), and catalase (CAT), and the non-enzymatic antioxidants include glutathione (GSH), ascorbate (AsA), as well as polyphenols and vitamins in plants (Meloni et al., 2003; Apel & Hirt, 2004; Naeem et al., 2018). Moreover, the increased synthesis of osmolytes such as soluble protein and proline also have a role in reducing stress (Sarropoulou et al., 2012; Turk et al., 2014). Nevertheless, severe stress for a long time would bring serious damages and cells death may occur (Turk et al., 2014). Therefore various studies have been conducted to improve crop resistance and to minimize the negative impact of various abiotic stresses, including drought stress. Different agronomic strategies have been adopted for this purpose. One of the innovative strategies is the use of plant bio-stimulators or phytohormones to improve the adaptability and protection of plants against unfavorable environmental conditions. Different plant bio-stimulators are reported to have positive effects on various physiological processes of the plants (Singh & Usha, 2003; Sarropoulou et al., 2012; Cui et al., 2017; Kamran et al., 2018).

Melatonin (*N-acetyl-5-methoxytryptamine*) is an important animal hormone and a natural indoleamine compound. It has an important role in numerous vital life processes, including the regulation of circadian rhythm, seasonal reproduction, antioxidant activity, immunological enhancement, and sleep (Hardeland et al., 2012; Jiang et al., 2016; Reiter, Tan & Sharma, 2018). Relatively “new” in plant biology, melatonin has gained a remarkable interest in the research community due to its broad dissemination in the biological kingdom and its possible involvement in many plants physiological processes. However, the regulatory and functional roles of melatonin in plants is still not fully understood, although melatonin has been implicated in improving plant growth and protection against various abiotic stresses in different crops (Jiang et al., 2016; Martinez et al., 2018; Lee & Back, 2018; Liang et al., 2019). Several studies have reported melatonin as a broad-spectrum antioxidant with a high capacity and efficiency for ROS scavenging (Tan et al., 2000; Tan et al., 2015). It has also been reported that melatonin interacts with the cell signaling mechanisms by activating redox-sensitive regulatory pathways and perhaps has a higher antioxidant capacity, and highly effective, even at low concentrations, in protecting against oxidative stress (Tan et al., 2015; Martinez et al., 2018). Presently, there is still an active discussion regarding the function of melatonin as a growth regulator and/or an antioxidant (Fleta-Soriano et al., 2017; Arnao & Hernández-Ruiz, 2019).

Many previous studies have reported that application of melatonin could stimulate root growth, promote seed germination and enhance photosynthetic characteristics in various crops (Martinez et al., 2018; Liang et al., 2019). The application of melatonin has been reported to not only encourage the germination of seedling and plant growth but also improve the plant stress resistance. A few studies also reported that exogenous application of melatonin could enhance stress tolerance in many crops by improving the growth in watermelon and cucumber under salinity stress (Li et al., 2017; Wang et al., 2016), in kiwi seedlings under drought stress (Liang et al., 2019), in *Arabidopsis thaliana* against endoplasmic reticulum stress (Lee & Back, 2018) as well as in cucumber under heat stress (Wang et al., 2016), linking the role of melatonin abiotic stress responses. In addition, the interaction of melatonin with ROS creates a series of derivatives compounds through a cascade reaction, all of them with high antioxidant capacity as well (Tan et al., 2015; Martinez et al., 2018; Reina et al., 2018).

However, knowledge about how melatonin modulates the physiological and biochemical changes in maize under drought stress remains elusive. In addition, available information regarding the precise concentration of melatonin for its application method in maize crop is limited, and thus warrants in-depth investigation. Therefore, the present study was focused on determining the possible effects of exogenous application of melatonin on physiological, biochemical and growth changes in maize seedling exposed to drought stress and exploring an optimal concentration for foliar application and soil drench. We mainly focused on the photosynthetic capacity and the oxidative system including reactive oxygen species accumulation and antioxidant enzymes, soluble proteins and proline content. The study will contribute to elucidate the mitigating effects of melatonin on drought-induced oxidative damages.

## Material and methods

### Plant material and experimental treatments

A pot experiment was conducted during April-June 2018 in the greenhouse at the Institute of Water Saving Agriculture Experimental Station of Northwest A&F University, Yangling, China. Maize (*Zea mays* L.) cv. Zhengdan 958, a widely cultivated variety in northwestern China was used in the experiment. Healthy maize seeds were first disinfected with sodium hypochlorite solution for 15 min and then thoroughly washed with distilled water several times. Six maize seeds were planted in each plastic pot (20 cm height, 18 cm diameter), filled with 4.5 kg soil (a mixture of farmland topsoil and compost 2:1 w/w). The pots were arranged in randomized complete block design and placed in a glass shed under natural light. The soil moisture in pots was maintained at 85–90% of FC for 20 DAS (days after sowing, V3 stage) and four maize seedlings with the same growth behavior were sustained in each pot. Afterward, a natural progressive drought was imposed by withholding watering (40–45% FC) throughout the experiment, based on daily measurements of pot weight. Melatonin was applied twice (25 and 30 DAS, V5 stage), as a foliar spray or soil drench. The treatments for foliar spray were: (1) FCK1: normal watering without melatonin, (2) FCK2: drought treatment without melatonin, (3) FM1: drought treatment with 25 µM melatonin, (4) FM2: drought treatment with 50 µM melatonin, (5) FM3: drought treatment with 75 µM melatonin, (6) FM4: drought treatment with 100 µM melatonin. The same treatments for melatonin were applied in drench application includes: (1) DCK1: normal watering without melatonin, (2) DCK2: drought treatment without melatonin, (3) DM1: drought treatment with 25 µM melatonin, (4) DM2: drought treatment with 50 µM melatonin, (5) DM3: drought treatment with 75 µM melatonin, (6) DM4: drought treatment with 100 µM melatonin. All the samplings and measurements were carried out at 50 DAS (V7 stage) for the various morphological, physiological and biochemical characteristics.

### Sampling and measurement

#### Plant growth attributes

Plants from each group were sampled and separated into roots, shoot and leaves to determine its fresh biomass. The root length was measured by root image analysis using WinRHIZO 2003a software (Regent Instruments, Quèbec, Canada). The stem diameter was measured using a digital Vernier caliper and plant height was measured with a tape meter. The plant samples were then oven-dried at 75 °C until reached a constant weight and its dry weight was determined. The leaf area (LA) was estimated by measuring maximum leaf length (LL) and maximum leaf width (LW) by using a ruler. Leaf area (LA) = LL × LW × 0.75.

Whereas 0.75 was the correction factor, and the leaf area was expressed as leaf area per plant.

#### Determination of leaf relative water content (RWC)

The leaf relative water concentration (RWC) was estimated by following Turk & Erdal (2015). The fresh weight (FW) of the seventh leaf was measured immediately after sample harvesting. After that leaf segments were immersed in distilled water overnight and its turgid weight (TW) was determined. The leaf samples were then oven-dried at 75 °C to obtain dry weight (DW). The relative water content (RWC) was calculated by using the following formula; RWC = [(FW − DW)∕(TW − DW)] × 100. Each treatment included three replicates.

#### Measurement of chlorophyll and carotenoids

The chlorophyll and carotenoid contents were measured by following the method of Arnon (1949). Briefly, 0.1 g of fresh leaf samples were sliced and placed in glass test tubes and 10 mL of 80% acetone was added to each tube and placed in a dark place until the complete discoloration of the leaves samples. The extract was centrifuged and the absorbance of the supernatant was measured at 645, 663, and 440 nm for determination of chlorophyll “a”, “b” and carotenoid using a UV-spectrophotometer, respectively.

#### Photosynthetic gas exchange

Gas exchange parameters (photosynthetic rate, stomatal conductance, intercellular CO_2_ concentration, and transpiration rate) were determined with a portable photosynthesis system LI- 6400XT (LI-COR, Biosciences, Lincoln, NE, USA). For determination of photosynthetic parameters, the top fully opened leaf was sampled at the V7 stage, between 9:00 and 11:00 AM. Each treatment was replicated three times.

#### Determination of antioxidant enzyme activity

For the determination of antioxidant enzymes activities, 0.5 g of leaf sample was homogenized in five mL of pre-cooled phosphate buffer (pH 7.6), containing 1 mM EDTA, and 4% (w/v) PVP, incubated at 4 °C for 10 min. After incubation, the homogenate was centrifuged (12, 000 × g) for 15 min at 4 °C, and the supernatant was used for subsequent estimation of enzymes. The enzymes activities were expressed as U g^−1^ FW.

Superoxide dismutase (SOD) activity was estimated based on the method of Giannopolitis & Ries (1977). The reaction mixture included 50 mM phosphate buffer (pH 7.6), 13 mM methionine, 750 mM NBT, 4 µM riboflavin, 0.1 mM EDTA mixed with 0.2 mL of the enzyme solution. The photochemical reduction of NBT was measured at 560 nm. One unit of SOD was defined as the amount of enzyme required that produced 50% inhibition of NBT reduction under assay conditions. The specific SOD activity was determined by using below formula and expressed as (U g^−1^FW).

\begin{eqnarray*}\text{SOD activity}({\text{U g}}^{-1}\text{FW})=(\Delta {\text{A}}_{560}\times \text{Vt})/(\text{W}\times \text{Vs}\times 0.05\times \text{t}) \end{eqnarray*}

ΔA_560_ is the change in absorbance, Vt is the total volume of the reaction mixture, W is the sample fresh weight, Vs is the volume of the crude enzyme, and t is the reaction time (min).

Peroxidase (POD) activity was assayed according to Cakmak & Marschner (1992). The reaction mixture contained 50 mM phosphate buffer (pH 7.0), 16 mM guaiacol, mixed with the 0.2 mL of enzyme extract followed by the addition of 10 mM H_2_O_2_. The absorbance of the mixture was measured at 470 nm.

\begin{eqnarray*}\text{POD activity}({\text{U g}}^{-1}\text{FW})=(\Delta {\text{A}}_{470}\times \text{Vt})/(\text{W}\times \text{Vs}\times 0.01\times \text{t}) \end{eqnarray*}

ΔA_470_ represents the time for the change in absorbance, Vt is the total volume of the reaction mixture, W is the sample fresh weight, Vs is the volume of the crude enzyme, and t is the reaction time (min).

Ascorbate peroxidase (APX) activity was measured following the method of Nakano & Asada (1981). The reaction mixture contained 50 mM phosphate buffer (pH 7.0), 0.1 mM EDTA, 0.5 mM AsA, 1.0 mM H_2_O_2_mixed with 0.2 mL crude enzyme extract. The change in absorbance of the mixture was measured at 290 nm using the formula.

\begin{eqnarray*}\text{APX activity}({\text{U mg}}^{-1})=(\Delta {\text{A}}_{290}\times \text{Vt})/(2.8\times \text{M}\times \text{V}\times \text{t}) \end{eqnarray*}

ΔA_290_ is the change in absorbance during 30 s, V is the volume of the crude enzyme, 2.8 mM^−^ cm^−^ is the extinction coefficient, M is mass of fresh materials, and t is the reaction time.

Catalase (CAT) activity was estimated by following the Havir & Mchale (1987). The reaction mixture contained 50 mM phosphate buffer (pH 7.0) and 12.5 mM H_2_O_2_ mixed with 0.2 mL of enzyme extract. The absorption of the mixture was measured at 240 nm.

\begin{eqnarray*}\text{CAT}({\text{U mg}}^{-1})=(\Delta {A}_{240}\times \text{Vt})/(\text{W}\times \text{Vs}\times 0.01\times \text{t}) \end{eqnarray*}

ΔA_240_ is the change in absorbance, Vt is the total volume of extracted enzyme solution, W sample fresh weight, t is reaction time, and Vs is the volume of crude enzyme extract.

#### Soluble protein and proline measurement

Soluble protein content was estimated by following Ashraf & Iram (2005). Briefly, 0.5 g leaf samples were homogenized with 10 mL phosphate buffer (pH 7.0) and centrifuged at 15, 000 × g for 20 min. The extract was treated with SERVA Blue-G and the absorbance was measured at 570 nm using a standard curve.

\begin{eqnarray*}\text{Soluble protein content}({\text{mg g}}^{-1}\text{FW})=(\text{C}\times \text{Vt})/(\text{W}\times \text{Vs}\times 1000) \end{eqnarray*}

Where C represents the standard curve obtained in the protein content (µg), Vt is the total volumes of the reaction mixture, Vs is the volume of supernatant, and W is the sample fresh weight.

Proline content was determined according to the procedure described by Tiwari et al. (2010). 0.5 g fresh samples were homogenized in 10 ml aqueous sulfosalicylic acid (3%) and centrifuged at 10, 000 × g for 15 min. Two mL of extract was treated with two mL of glacial acetic acid and two mL acid ninhydrin and then with four mL of toluene. The absorbance of the colored solutions was determined at 520 nm, using toluene for a blank. Proline concentration was determined from a standard curve and calculated as follows.

\begin{eqnarray*}\text{Proline concentration}({\text{\mathrm{\mu} g g}}^{-1}\text{FW})=({\text{OD}}_{520}-\text{blank})/\text{slope}\times \text{Vt/Vs}\times 1/\text{FW} \end{eqnarray*}

Where: OD_520_ absorbance of the extract, blank (expressed as absorbance) and slope (expressed as absorbance ⋅nmol^−1^ ) are determined by linear regression, Vt is the total volume of the extract, Vs is the volume of crude extract used, and FW is the plant material.

#### Quantification of malondialdehyde (MDA) and H_**2**_**O**_**2**_ concentration

H_2_O_2_ content was determined by following the method of Velikova, Yordanov & Edreva (2000). The reaction mixture contained 2.5 mM phosphate buffer (pH 7.0) and 500 mM potassium iodide mixed with 200 µL of enzymes supernatant. The absorbance of the mixture was determined at 390 nm. The difference in absorbance between the blank and the other aliquot was used to calculate the concentration of H_2_O_2_ using an extinction coefficient of 39.4 µM^−1^ cm^−1^.

MDA as an end product of lipid peroxidation was measured according to the method of (Cakmak & Marschner, 1992), with slight modifications. The MDA reaction mixture including 500 µL of supernatant, 0.65% (w/v) thiobarbituric acid (TBA) in 20% trichloroacetic acid (TCA)] was boiled for 30 min, and immediately cooled to stop the reaction. The mixture was then centrifuged at 10,000× g for 10 min. The absorbance of the mixture was measured at 532 nm. The nonspecific absorption at 600 nm was subtracted from the absorbance data

\begin{eqnarray*}\text{MDA content}({\text{\mathrm{\mu} mol g}}^{-1}\text{FW})=(\text{C}\times \text{V / W}) \end{eqnarray*}

Whereas C is the difference in nonspecific absorption between 600 and 532 nm, V is the volume of the extract and W is the fresh weight of the sample.

### Statistical analysis

The experimental data were organized and processed using Microsoft Excel and are presented as ± SD. Data were analyzed by using SPSS 20.0 statistical software. One-way analysis of variance (ANOVA) was carried out to find out the significant difference among the treatment means at *p* < 0.05.

## Results

### Effect of melatonin on root length and diameter

The maize seedlings showed a drastic decline in root length and diameter under drought stress, compared to well water (Table 1 and Fig. S1). The application of melatonin as a foliar spray and soil drench facilitated the root elongation of maize seedlings and was significantly greater than that in respective drought stress (FCK2 & DCK2). The root length showed a substantial expansion with the increase in the concentration of melatonin as a foliar application, the highest root length was observed in FM4 treatment (Table 1 and Fig. S1). Compared to FCK2, the root length of FM1, FM2, FM3, and FM4 treatments were greater by 9.9, 26.0, 34.3, and 43.5%, respectively. In soil drench application, root length showed a significant increase in melatonin treatments, regardless of its concentration and no significant differences were perceived among the various melatonin concentrations i.e., DM1, DM2, DM3, and DM4 (Table 1 and Fig. S1). Likewise, the average root diameter presented a progressive increase with the melatonin concentration and we found that the average root diameter was evidently greater in FM4 and DM4 treatments, which was greater by 34.9 and 19.0%, compared to FCK2 and DCK2, respectively.

**Table 1:** Effects of different melatonin treatments on various morphological growth indicators in maize seedlings under drought stress.

Melatonin treatments^a^		Root length (cm plant^−1^)	Root diameter (mm)	Root fresh weight (g)	Root dry weight (g)	Shoot fresh weight (g)	Shoot dry weight(g)
**Foliar spray**	FCK1	811.0 ± 18.76a	1.96 ± 0.05b	8.01 ± 0.33a	0.76 ± 0.07a	16.13 ± 0.19a	1.92 ± 0.02a
	FCK2	562.3 ± 32.51e	1.53 ± 0.04e	5.42 ± 0.46d	0.41 ± 0.03c	9.12 ± 0.74e	0.93 ± 0.04e
	FM1	618.0 ± 21.75d	1.59 ± 0.04e	6.40 ± 0.60c	0.50 ± 0.05c	10.62 ± 0.78d	1.12 ± 0.06d
	FM2	708.5 ± 15.13c	1.68 ± 0.02d	6.89 ± 0.11bc	0.61 ± 0.06b	12.43 ± 0.57c	1.44 ± 0.16c
	FM3	755.0 ± 27.12b	1.80 ± 0.03c	7.62 ± 0.34ab	0.69 ± 0.03ab	13.01 ± 0.78c	1.67 ± 0.15b
	FM4	807.0 ± 15.43a	2.05 ± 0.08a	7.93 ± 0.28a	0.74 ± 0.06a	14.59 ± 0.48b	1.75 ± 0.12ab
**Soil Drench**	DCK1	797.6 ± 08.28a	1.90 ± 0.10a	6.69 ± 0.16a	0.63 ± 0.01a	14.30 ± 0.47a	1.62 ± 0.01a
	DCK2	586.7 ± 24.46c	1.56 ± 0.04c	4.74 ± 0.58c	0.36 ± 0.04d	9.50 ± 0.21e	0.97 ± 0.09d
	DM1	730.1 ± 23.98b	1.69 ± 0.03b	5.55 ± 0.32b	0.43 ± 0.02cd	11.18 ± 0.64d	1.20 ± 0.11c
	DM2	744.1 ± 25.04b	1.67 ± 0.03b	5.78 ± 0.34b	0.48 ± 0.05bc	14.10 ± 0.66a	1.58 ± 0.08ab
	DM3	749.6 ± 17.35b	1.69 ± 0.02b	6.03 ± 0.18b	0.54 ± 0.06b	13.28 ± 0.21b	1.47 ± 0.04b
	DM4	746.4 ± 25.58b	1.85 ± 0.04a	6.05 ± 0.17b	0.53 ± 0.07b	12.58 ± 0.44c	1.29 ± 0.08c

**Notes.**

a For Foliar application, FCK1 indicate well-watered, FCK2, drought stress; FM1, FM2, FM3, and FM4 indicates melatonin application at the rate of 25, 50, 75, 100 M, respectively. For soil drench application, DCK1 indicates well-watered, DCK2, drought stress; DM1, DM2, DM3, and DM4 indicates melatonin application at the rate of 25, 50, 75, 100 M. Data are shown as the mean ± S.D. (*n* = 3). Different letters in the same column indicate a significant difference in particular series at *P* < 0.05 as determined by the LSD test.

### Effects of melatonin on the biomass of maize seedlings

Melatonin application significantly improved the growth characteristics of maize seedlings under drought stress. As shown in Table 1, fresh and dry root and shoot biomass of maize seedlings were markedly lower under drought stress as compared to well-watered, while the negative effects of drought stress on biomass was significantly alleviated and were gradually increased with increase in the concentration of melatonin under the foliar application. Fresh weight and dry weight of the roots in FM1, FM2, FM3, and FM4 treatments were higher than that in FCK2, with 18.0 and 23.1%, 27.0 and 51.3%, 40.5 and 70.1%, 46.2 and 82.7% increase respectively. Likewise, root fresh and dry weight showed a similar trend as that of root length in melatonin drench application. Root fresh and dry weight in all the treatments were greater than control. However, it was not significant statistically.

Shoot fresh and dry biomass accumulation in melatonin-treated plants showed significant improvement compared to control plants under drought stress. In case of foliar application, the highest concentration of melatonin (FM4) markedly improved shoot fresh biomass by 60.1%, and dry biomass by 88.4% compared with FCK2 (Table 1). While in case of drench application, unlike root biomass, the shoot biomass was expressed differently among the various melatonin treatments. Shoot fresh and dry biomass exhibited an initial increase with the concentration of melatonin and then declined considerably at higher concentrations. DM2 treatment exhibited the most promising effect on shoot biomass, followed by DM3. It significantly enhanced shoot fresh and dry biomass by 48.5% and 39.8%, 62.9% and 52.1%, compared with DCK2, respectively.

### Stem diameter and plant height

Plant height and stem diameter were evaluated in maize seedling treated with melatonin under drought stress. Melatonin-treated seedlings were appeared to be taller and had thicker stems as compared to control treatment under drought stress (Table 2). In foliar application, FM4 exhibited greater stem diameter and plant height and was greater by 71.4 and 38.5% than that of FCK2. However, in soil drench application, higher stem diameter and plant height were achieved by DM2 treatments and was greater by 73.4 and 40.1% than DCK2. The concentrations of melatonin, i.e., DM3 and DM4 showed a relative decrease in stem diameter and plant height than that in the DM2 treatment, showing the inhibitory effects of higher melatonin concentrations, it allows us to optimize melatonin concentration, which plays a vital role in drought stress management and crop improvement.

**Table 2:** Effects of different melatonin treatments on various morphological indicators and photosynthetic pigments in maize seedlings under drought stress.

Melatonin treatments^a^		Stem diameter (mm)	Plant height (cm)	RWC (%)	Leaf area (cm^2^ plant^−1^)	Chlorophyll a (mg g^−1^ FW)	Chlorophyll b (mg g^−1^ FW)	Carotenoid (mg g^−1^ FW)
**Foliar Spray**	FCK1	9.51 ± 0.21a	63.5 ± 0.74a	94.2 ± 3.08a	486.3 ± 6.83a	2.92 ± 0.21ab	0.68 ± 0.03a	0.35 ± 0.02c
	FCK2	4.97 ± 0.61e	42.5 ± 1.67e	71.0 ± 1.21e	341.4 ± 8.20d	1.09 ± 0.09d	0.29 ± 0.02e	0.23 ± 0.01e
	FM1	6.05 ± 0.12d	49.0 ± 1.85d	77.6 ± 3.20d	383.6 ± 6.62c	1.37 ± 0.12d	0.30 ± 0.01e	0.28 ± 0.02d
	FM2	7.07 ± 0.36c	55.1 ± 2.18c	83.6 ± 1.62c	407.3 ± 6.68b	1.93 ± 0.13c	0.38 ± 0.01d	0.29 ± 0.01d
	FM3	8.02 ± 0.51b	58.3 ± 1.87b	86.7 ± 1.81bc	432.1 ± 6.75b	2.65 ± 0.34b	0.47 ± 0.06c	0.38 ± 0.02b
	FM4	8.51 ± 0.81b	58.8 ± 2.92b	89.7 ± 1.07b	445.5 ± 4.44b	3.02 ± 0.37a	0.61 ± 0.03b	0.42 ± 0.01a
**Soil Drench**	DCK1	9.00 ± 0.16a	64.1 ± 1.01a	91.0 ± 1.29a	502.5 ± 6.81a	3.08 ± 0.16a	0.63 ± 0.03a	0.37 ± 0.01b
	DCK2	5.16 ± 0.41d	43.6 ± 1.25e	68.3 ± 1.69e	329.4 ± 10.26e	1.24 ± 0.22c	0.31 ± 0.04d	0.25 ± 0.01d
	DM1	6.68 ± 0.72c	53.8 ± 1.94d	78.6 ± 1.95d	373.2 ± 8.19d	2.23 ± 0.36b	0.40 ± 0.02c	0.31 ± 0.01c
	DM2	8.95 ± 0.33a	61.1 ± 1.98b	85.8 ± 1.05b	440.1 ± 9.94c	2.91 ± 0.15a	0.57 ± 0.06ab	0.37 ± 0.02b
	DM3	8.74 ± 0.26a	59.4 ± 1.16bc	84.6 ± 1.75bc	466.3 ± 9.69b	2.56 ± 0.45ab	0.55 ± 0.05b	0.41 ± 0.01a
	DM4	7.94 ± 0.13b	57.1 ± 0.53c	82.6 ± 1.37c	379.7 ± 8.19d	2.28 ± 0.41b	0.56 ± 0.04b	0.43 ± 0.02a

**Notes.**

a For Foliar application, FCK1 indicate well-watered, FCK2, drought stress; FM1, FM2, FM3, and FM4 indicates melatonin application at the rate of 25, 50, 75, 100 M, respectively. For soil drench application, DCK1 indicates well-watered, DCK2, drought stress; DM1, DM2, DM3, and DM4 indicates melatonin application at the rate of 25, 50, 75, 100 M. Data are shown as the mean ± S.D. (*n* = 3). Different letters in the same column indicate a significant difference in particular series at *P* < 0.05 as determined by the LSD test.

### Leaf relative water content

Leaf relative water content (RWC) are the main indicators that show water status and survival ability of the plants under stress condition. A significant decrease in RWC of drought-stressed maize seedlings was observed as compared to well-watered plants. On the other side, there was a significant improvement in RWC of melatonin-treated maize seedlings under drought stress condition (Table 2). The most substantial increase in RWC was observed in FM4 and DM2 treatments and was greater by 26.4 and 25.5%, compared to the respective CK2 treatment.

### Chlorophyll and carotenoid content

Our results showed that drought stress significantly decreased the chlorophyll and carotenoids concentration when compared with well-water maize seedling. However, melatonin application, regardless of its application method, significantly increased the chlorophyll of maize seedlings under drought stress (Table 2). Among the foliar treatments of melatonin, FM4 showed the most promising effects, which increased Chl a, Chl b, and carotenoid by 183.1%, 113.2%, and 80.4%, respectively, compared to FCK2. However, among soil drench treatments, greater Chl a was recorded in DM2 treatment and was greater by 135.2% than DCK2. Higher melatonin concentrations (DM3 and DM4) employed a negative influence on the content of Chl a, provoking a steady decrease, compared to the DM2 treatment. Likewise, the maximum content of chlorophyll b was also calculated for DM2 (80.6% greater than DCK2) treatment, but no significant differences among DM2, DM3, and DM4 treatments. On the other hand, higher carotenoid concentration was expressed in DM4 treatment and was greater by 69.1%, followed by DM3, greater by 63.4% than DCK2 (Table 2).

### Effect of melatonin on leaf gas exchange

**Figure 1:**
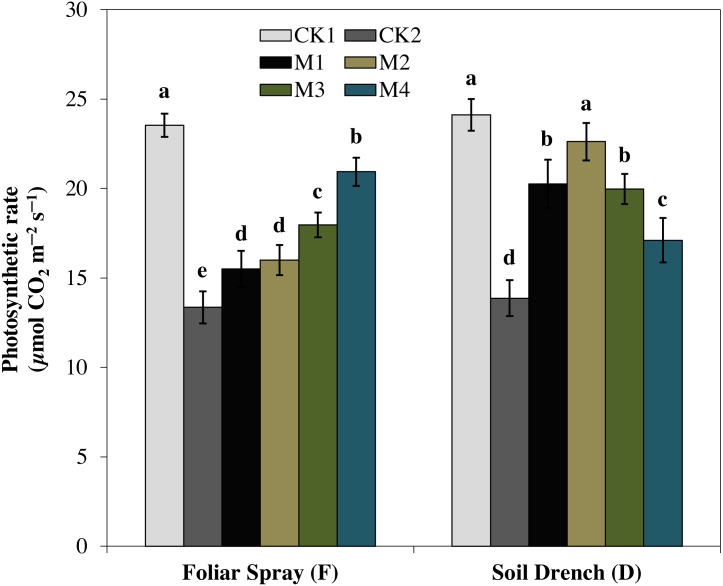
Effects of melatonin on net photosynthetic rate in maize seedlings under drought stress. CK1, normal watering without melatonin; CK2, drought treatment without melatonin; M1, drought treatment with 25 µM melatonin; M2, drought treatment with 50 µM melatonin; M3, drought treatment with 75 µM melatonin; M4, drought treatment with 100 µM melatonin. Both foliar spray and soil drench use the sameconcentrations of melatonin. Vertical bars represents ± S.D. (*n* = 3). Different letters indicate significant differences as determined by the LSD test (*p* < 0.05).

**Figure 2:**
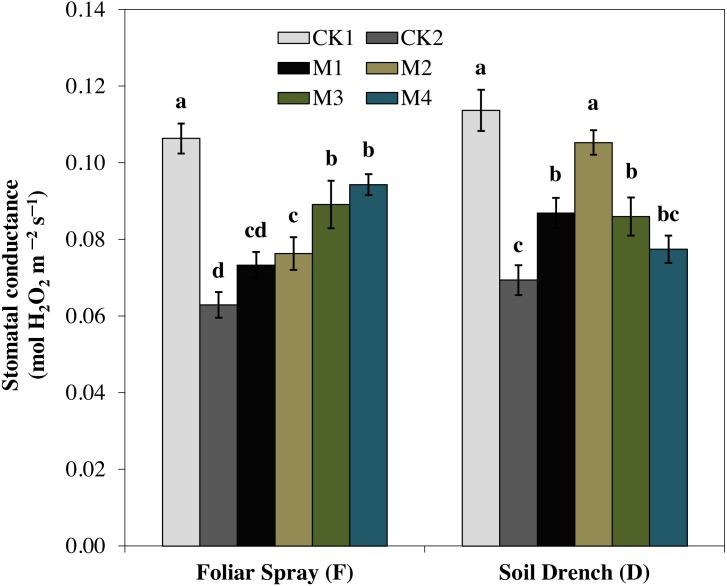
Effects of melatonin on the stomatal conductance in maize seedlings under drought stress. The treatments names are the same as those described in Fig. 1. Both foliar spray and soil drench use the same concentrations of melatonin. Vertical bars represents ± S.D. (*n* = 3). Different letters indicate significant differences as determined by the LSD test (*p* < 0.05).

**Figure 3:**
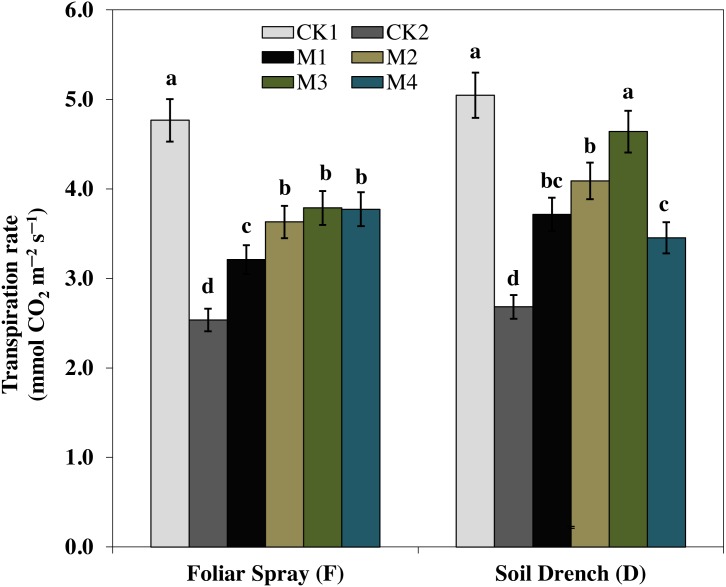
Effects of melatonin on transpiration rate in maize seedlings under drought stress. The treatments names are the same as those described in Fig. 1. Both foliar spray and soil drench use the same concentrations of melatonin. Vertical bars represents ± S.D. (*n* = 3). Different letters indicate significant differences as determined by the LSD test (*p* < 0.05).

One of the prime impacts of drought stress is on the physiological process of photosynthesis (Pn). As shown in (Figs. 1–3), drought stress caused a severe decrease in net photosynthetic rate and stomatal conductance (Gs) values of maize seedlings. However, treatment with various melatonin concentrations obviously alleviated drought stress-induced reduction in leaf Pn and Gs. The FM1, FM2, FM3, and FM4 treatments significantly increased Pn by 16.1, 19.8, 34.5, 56.8%, while that of Gs values were increased by 16.5, 21.2, 41.6, and 49.8%, compared to FCK2, respectively. Application of melatonin as a soil drench to drought-stressed seedling also showed a remarkable difference in Pn and Gs values, however, Pn and Gs were markedly improved at lower concentrations (DM1 and DM2), and later significantly inhibited at the highest concentration of melatonin (DM3 and DM4). The Pn values of DM1 and DM2 was greater by 46.1 and 63.1%, while Gs values were greater by 25.2 and 51.8%, compared with DCK2, respectively. The transpiration rate was also considerably higher in all melatonin-treated plants than untreated control under drought stress (Fig. 3).

### Effects of melatonin on antioxidant enzymes

**Figure 4:**
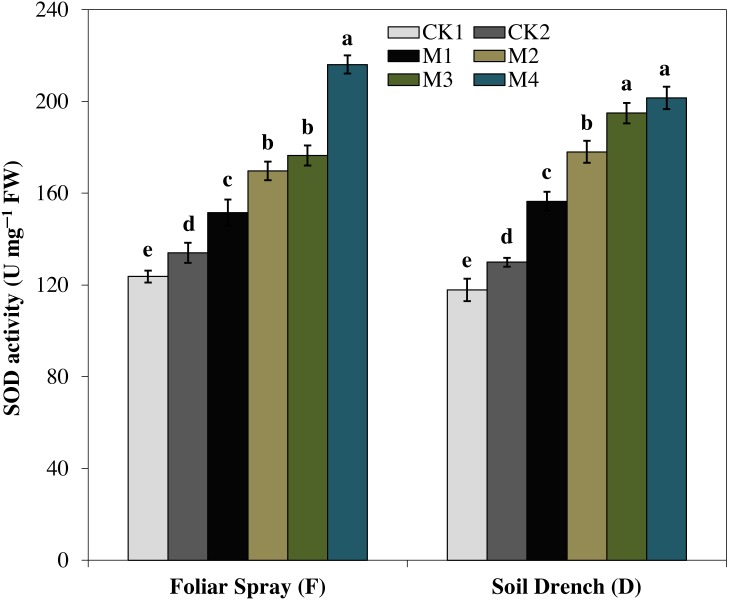
Effects of melatonin on superoxide dismutase (SOD) activity in maize seedlings under drought stress. The treatments names are the same as those described in Fig. 1. Both foliar spray and soil drench use the same concentrations of melatonin. Vertical bars represents ± S.D. (*n* = 3). Different letters indicate significant differences as determined by the LSD test (*p* < 0.05).

The activities of antioxidant enzymes were markedly enhanced by the application of melatonin in comparison with untreated maize seedlings subjected to drought stress. However, the different melatonin treatments showed a quite different pattern for improving the activities of different antioxidant enzymes. For instance, superoxide dismutase (SOD) activity showed an increase with increasing melatonin concentrations, both under the foliar spray and soil drench application (Fig. 4). The FM1, FM2, FM3, and FM4 treatments expressed an increase in SOD activity by 13.1, 26.6, 31.6, and 61.2%, while that of DM1, DM2, DM3, and DM4 treatments were increased by 20.5, 39.5, 50.1, and 55.1%, as compared to their corresponding control under drought (FCK2 and DCK2), respectively.

**Figure 5:**
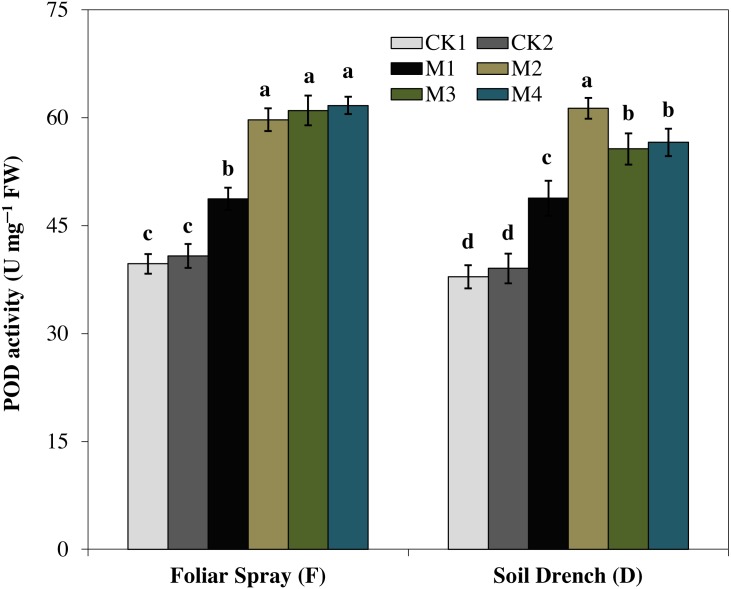
Effects of melatonin on peroxidase (POD) activity in maize seedlings under drought stress. The treatments names are the same as those described in Fig. 1. Both foliar spray and soil drench use the same concentrations of melatonin. Vertical bars represents ± S.D. (*n* = 3). Different letters indicate significant differences as determined by the LSD test (*p* < 0.05).

The activity of peroxidase (POD) showed a totally different increasing pattern (Fig. 5). Under foliar spray, the POD activity initially increased with increasing concentration of melatonin reached a maximum in FM2 treatment (greater by 46.5% than FCK2). A further increase in the concentration of melatonin (FM3 and FM4) was not statically significant from that of FM2. In soil drench application, the POD activity attained the highest single peak curve under DM2 treatment and was 57.1% greater than DCK2. The higher concentrations of melatonin (DM3 and DM4) slightly inhibited the enzyme activity compared to that of FM2, but the enzyme activity in these treatments was still higher than the untreated control (Fig. 5).

**Figure 6:**
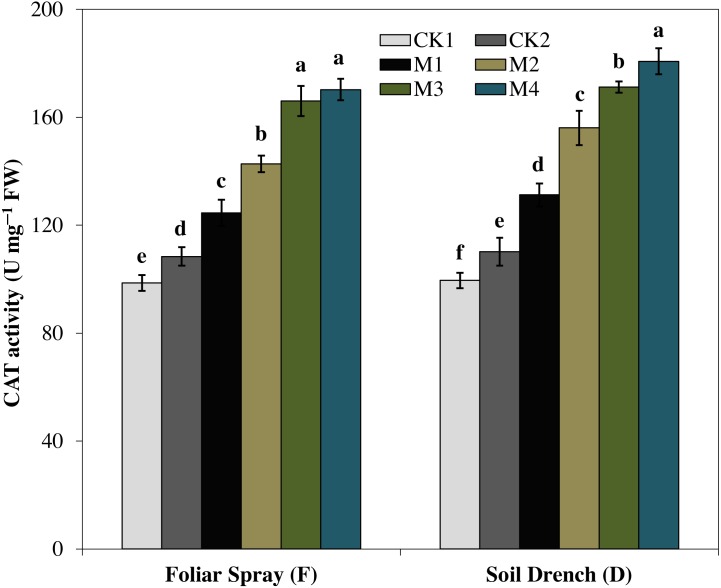
Effects of melatonin on catalase (CAT) activity in maize seedlings under drought stress. The treatments names are the same as those described in Fig. 1. Both foliar spray and soil drench use the same concentrations of melatonin. Vertical bars represents ± S.D. (*n* = 3). Different letters indicate significant differences as determined by the LSD test (*p* < 0.05).

The activity of catalase (CAT) enzyme presented an increasing pattern similar to SOD activity. The CAT activity revealed an increasing tendency with the concentration of melatonin and reached to single peak curve in FM4 and DM4 treatments. Compared to the untreated control (FCK2 and DCK2), the enzyme activity of FM4 and DM4 treatments were increased by 57.1 and 64.0%, respectively (Fig. 6).

**Figure 7:**
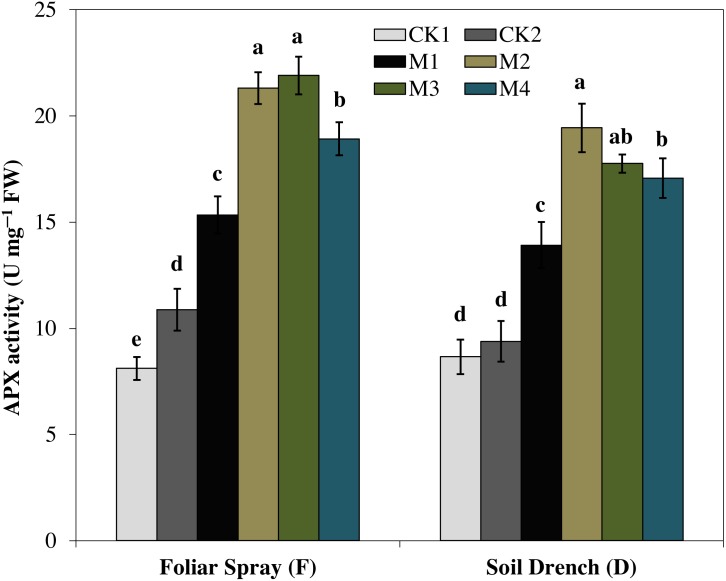
Effects of melatonin on ascorbate peroxidase (APX) activity in maize seedlings under drought stress. The treatments names are the same as those described in Fig. 1. Both foliar spray and soil drench use the same concentrations of melatonin. Vertical bars represents ± S.D. (*n* = 3). Different letters indicate significant differences as determined by the LSD test (*p* < 0.05).

Ascorbate peroxidase (APX) activity was evidently improved by melatonin treatments, which first increase with increasing concentration and then later decreased at the higher concentration under both foliar spray and soil drench application (Fig. 7). The maximum APX activity was observed in FM2 and DM2 treatments which elevated the APX activity of maize seedlings by 101.4 and 107.1%, in comparison with that of FCK2 and DCK2, respectively.

### Effects of melatonin on H_**2**_**O**_**2**_ and malondialdehyde

**Figure 8:**
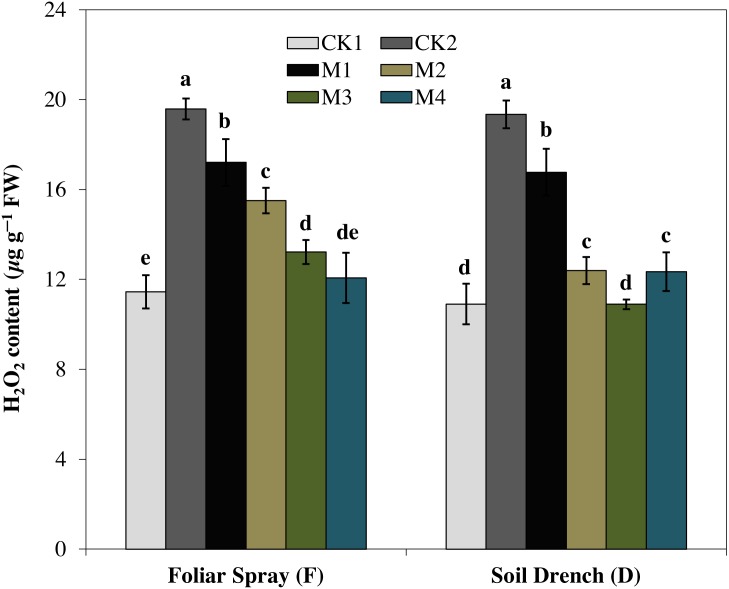
Effects of melatonin on H2O2 levels in maize seedlings under drought stress. The treatments names are the same as those described in Fig. 1. Both foliar spray and soil drench use the same concentrations of melatonin. Vertical bars represents ± S.D. (*n* = 3). Different letters indicate significant differences as determined by the LSD test (*p* < 0.05).

H_2_O_2_ is ROS produced by cellular metabolism and is an indicator of the ROS scavenging capacity of plants under stress. In the present study, H_2_O_2_ content was increased by 71.1% under FCK2 and 77.4% in DCK2 treatment, compared with that of FCK1 and DCK1, respectively. In contrast, the production of H_2_O_2_ was evidently reduced by melatonin treatments at variable degrees (Fig. 8). Regardless of the melatonin application method, the generation of H_2_O_2_ was steadily attenuated with an increase in the concentration of melatonin. The H_2_O_2_ content of the FM1, FM2, FM3, and FM4 treatments were 12.2, 20.8, 32.5, and 38.3% lower than FCK2, while in DM1, DM2, DM3, and DM4 treatments, the H_2_O_2_ contents were 13.3, 36.0, 43.7, and 36.2% lowered than DCK2, respectively.

**Figure 9:**
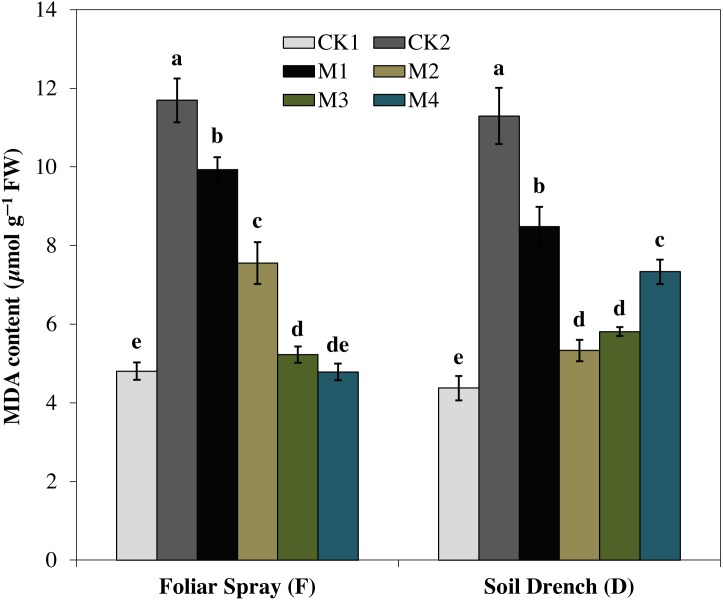
Effects of melatonin on malondialdehyde (MDA) content in maize seedlings under drought stress. The treatments names are the same as those described in Fig. 1. Both foliar spray and soil drench use the same concentrations of melatonin. Vertical bars represents ± S.D. (*n* = 3). Different letters indicate significant differences as determined by the LSD test (*p* < 0.05).

Correspondingly, the imposition of drought stress causes a considerable increase in MDA accumulation and was greater by 143.5% in FCK2 than FCK1, while increased by 158.3% in DCK2 than that in DCK1(Fig. 9). Supplying melatonin to the drought-stressed maize seedlings as a foliar spray and soil drench resulted in a reduction in the accumulation of MDA content. In comparison with the untreated control, the least MDA contents were observed for FM3 and FM4 treatments for melatonin foliar application, which were decreased by 55.3 and 59.1%, respectively. Regarding melatonin soil drench application, the DM2 and DM3 treatments evinced a decrease of 52.8 and 48.5% in the MDA contents, compared to the untreated control, respectively. Nevertheless, the highest concentration (DM4) showed a slight increase in MDA contents than that of DM2 and DM3 treatments (Fig. 9).

### Effects of melatonin on soluble protein and proline contents

**Figure 10:**
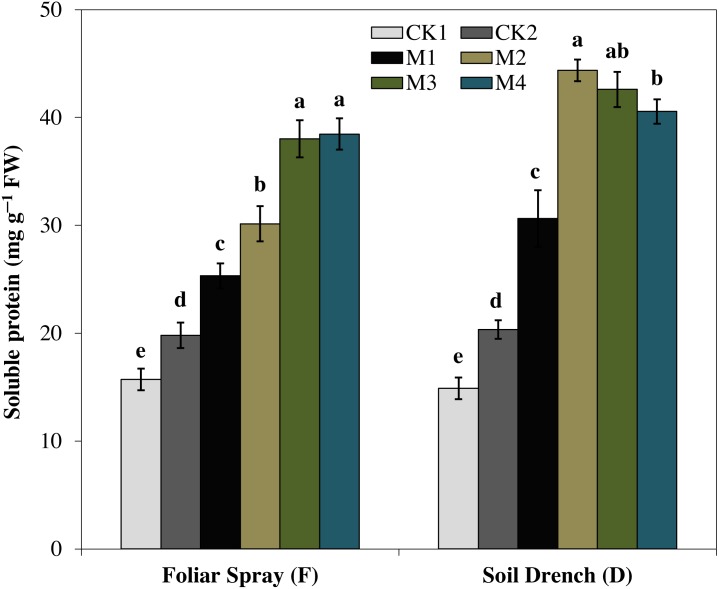
Effects of melatonin on soluble protein in maize seedlings under drought stress. The treatments names are the same as those described in Fig. 1. Both foliar spray and soil drench use the same concentrations of melatonin. Vertical bars represents ± S.D. (*n* = 3). Different letters indicate significant differences as determined by the LSD test (*p* < 0.05).

**Figure 11:**
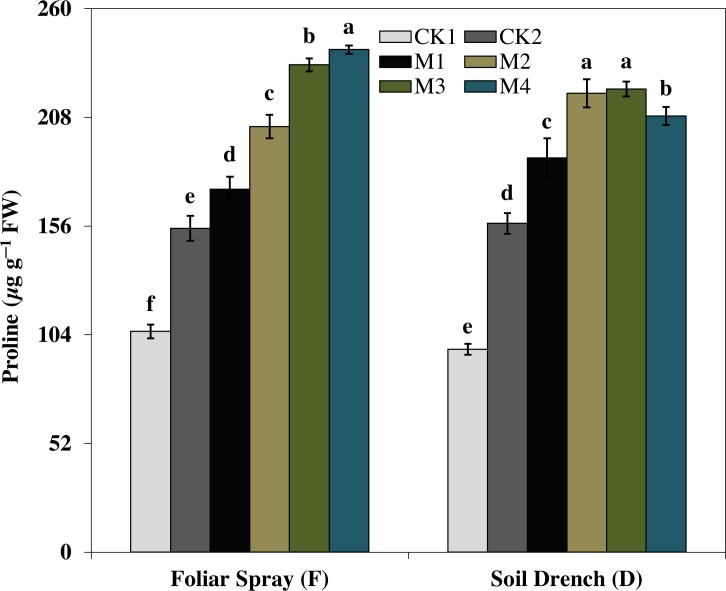
Effects of melatonin on proline content in maize seedlings under drought stress. The treatments names are the same as those described in Fig. 1. Both foliar spray and soil drench use the same concentrations of melatonin. Vertical bars represents ± S.D. (*n* = 3). Different letters indicate significant differences as determined by the LSD test (*p* < 0.05).

The results showed that the soluble protein (Fig. 10), and proline (Fig. 11), contents were reasonably increased after imposition of drought stress, showing the biochemical alteration of maize seedlings to drought stress. Nevertheless, the soluble protein and proline contents were further enhanced in the melatonin-treated plant at a variable degree. Soluble protein contents were significantly increased by 27.9% , 52.3% , 92.1% , 94.3% in FM1, FM2, FM3, and FM4 treatments, while the proline contents were increased by 121, 31.5, 50.5, and 55.3%, compared with FCK1, respectively. Likewise, the soluble protein contents showed a significant increase of 50.6% , 118.2% , 109.5% , and 99.4% in DM1, DM2, DM3, and DM4 treatments, and that of proline contents were increased by 121% , 31.5% , 50.5% , and 55.3% in DM1, DM2, DM3, and DM4, compared with DCK1, respectively.

## Discussion

Drought stress is well known for its inhibitory effects. It markedly decreases the growth and development of plant and ultimately reduce crop yield (Munns & Tester, 2008). Higher plants have developed various types of strategies to respond to stresses. Melatonin is considered to be involved in different stress responses (Szafrańska, Reiter & Posmyk, 2016). During our study, the protective role of melatonin was investigated in maize seedlings under drought stress. In the present study, the growth of maize seedlings was markedly inhibited after the plants were exposed to drought stress. However, the application of melatonin as foliar spraying and soil drench reduced the severity of drought-induced growth inhibition. It enhanced maize tolerance to drought stress in terms of promoting the plant’s growth. The treated plants had improved stem diameter and plant height, greater biomass of roots and shoot and thicker leaves with higher leaf area than those that received no melatonin (Tables 1–2). The increase in biomass accumulation and growth attributes is endorsed to the improved carbon assimilation due to enhanced photosynthetic capacity of the treated plants, which is in agreement with the previous study of Zeng et al. (2018). Therefore, our experiment indicated that melatonin application effectively enhanced the adaptability of seedlings to drought stress by ameliorating the suppression of growth characteristics caused by drought. Our results further confirmed the findings of the previous reports that application of melatonin alleviated drought stress and positively affected plant growth in kiwifruit seedlings (Liang et al., 2019), watermelon (Li et al., 2017), Pea (*Pisum sativum)* (Szafrańska, Reiter & Posmyk, 2016), and tomato plant (Martinez et al., 2018; Liang et al., 2019).

Remarkably, denser roots with greater root length and root diameter were observed in melatonin-treated plants than untreated control plants under drought stress (Table 1 and Fig. S1). It is likely that melatonin application increases the level of endogenous growth factors, which resulted in the formation of denser roots (Zeng et al., 2018). Melatonin-induced root growth is regulated by auxin-modulated physiological processes, affecting the uptake of water and initiating irreversible cell wall extension (Bleiss & Ehwald, 1993). The value-added root increase seems to be melatonin mediated enhanced adaptability of treated plants for more efficient acquisition of soil water and nutrients and to transport it to the aerial parts of plants. Hence, we concluded that melatonin plays an important role in strengthening maize seedlings roots grown under drought stress conditions. Previous studies have demonstrated that melatonin supplementation increases root growth in cherry (Sarropoulou et al., 2012), improved the vigor of cucumber roots under drought stress (Zhang et al., 2013), and increased root length and diameter in rape seedlings under salinity stress (Zeng et al., 2018).

Chlorophyll is an important pigment involved in photosynthesis and plays a key role in the absorption and transmission of light energy (Arnao & Hernández-Ruiz, 2009). Likewise, carotenoids are an important pigment, serves as photo-protectants and functions as safety valves releasing excess energy before it can damage plant cells (Szafrańska, Reiter & Posmyk, 2016). Drought stress causes a severe reduction in leaf area, degrades photosynthetic pigments, which undeniably impairs and reduce photosynthesis, directly affecting plant growth (Muller et al., 2014; Naeem et al., 2018; Liang et al., 2019). Application of exogenous melatonin preserves chlorophyll pigments and as a result improves photosynthesis in the plants grown under stress conditions (Arnao & Hernández-Ruiz, 2009; Wang et al., 2016; Cui et al., 2017). Previously, melatonin application has been reported to maintain more levels of chlorophyll pigments in cucumber seedlings under salt stress (Wang et al., 2016), apple leaves under salt stress (Li et al., 2012), and delayed senescing in barley leaves (Arnao & Hernández-Ruiz, 2009). Our results showed that the application of melatonin alleviates the negative effects of drought stress and increased leaf area per plant, improved the content of chlorophyll and carotenoid compared with the untreated control plants under drought stress (Table 2). These results indicated that a high concentration of melatonin treatment in leaves enhance the biosynthesis of chlorophyll pigments and reduce its decomposition, initiated due to drought stress. This increase in chlorophyll contents is partly contributed to improved photosynthetic gas exchange. Our results regarding improvement in chlorophyll contents due to exogenous melatonin application under drought stress are further confirmed by the previous findings of Sarropoulou et al. (2012), Zhang et al. (2017), and Fleta-Soriano et al. (2017).

Under drought stress, stomatal closure is one of the primary plant response to minimize water loss, accompanied by a remarkable decrease in stomatal conductance and consequently, stomatal limitation of photosynthesis (Meloni et al., 2003; Liu et al., 2013). However, previous studies reported that an optimum dose of melatonin application improved the stomatal functions by enabling plants to reopen their stomata under drought-stressed wheat, cucumber and Malus, suggesting a positive role of melatonin in stomatal regulation (Li et al., 2015; Li et al., 2017; Cui et al., 2017). The photosynthetic rate was improved by exogenous application of melatonin in watermelon under salt stress (Li et al., 2017), and in pea (*Pisum sativum*) under paraquat stress (Szafrańska, Reiter & Posmyk, 2016). In our experiment, we also observed that net photosynthesis rate was significantly lower in the untreated plant under drought stresses, undoubtedly due to stomatal limitations (Fig. 1). Melatonin treatments, however, found to escalate net photosynthetic rate and stomatal conductance, and cause a simultaneous increase in leaf relative water content. The effects of melatonin on various photosynthetic traits were highly dose-dependent (Figs. 1–3). The highest concentration of melatonin as a foliar spray and a moderate concentration as soil drench were more effective in improving photosynthesis under drought stress. Accordingly, the alleviation of stomatal limitation by melatonin contributed to enhancing the net photosynthetic rate under drought stress. Our results are in agreement with that of Cui et al. (2017), who reported a similar increase in photosynthetic rate and stomatal conductance of melatonin-treated wheat seedlings grown under stress conditions. Nevertheless, the melatonin treatments also resulted in an increase transpiration rate, possibly driven by the enhanced stomatal conductance to maintain a steady state of photosynthesis under drought stress, which is in line with the previous studies (Ye et al., 2016; Cui et al., 2017).

Oxidative damage is the primary consequence of environmental stresses due to excessive accumulation of (ROS) (Naeem et al., 2018). The severity of oxidative damage to the cell membrane by H_2_O_2_ as lipid peroxidation is commonly measured as MDA. To cope with the oxidative damage, plants have evolved an effective defense mechanism of antioxidants (enzymatic/non-enzymatic) including SOD, POD, CAT, APX (Wang et al., 2016; Cui et al., 2017). SOD is involved in the primary step of cellular defense and is a key enzyme regulating the O•^−2^ status in leaves. Additionally, CAT and APX regulated the H_2_O_2_ accumulation and reduce it to H_2_O. In the present study, the activities of antioxidant enzymes were lower in untreated control plants under drought stress (Figs. 4–7). Notably, the negative effect of drought stress was retreated by application of melatonin, which greatly upsurges the activities of these antioxidant enzymes. However, the different antioxidant enzymes showed a different increasing pattern with increase in the concentration of melatonin applied as a foliar spray and/or soil drench. Previous studies reported that melatonin application in various crops evidently enhanced their stress tolerance by protecting the photosynthetic apparatus, increasing their antioxidant capability, and improving the water-holding capacity (Wei et al., 2015; Cui et al., 2017; Fleta-Soriano et al., 2017). The application of exogenous melatonin significantly decreased the accumulation of H_2_O_2_ and lipid peroxidation level, evident by lower MDA contents (Figs. 8 and 9). This decrease in ROS and reduced accumulation of MDA is attributed partly to the enhanced activities of antioxidant enzymes that effectively eliminated the excessive ROS. On the other hand, melatonin is considered as a broad-spectrum antioxidant and free radical scavenger (Tan et al., 2000; Dawood & El-Awadi, 2015), and hence, might directly eliminate ROS when produced under stressed conditions. The previous studies of Szafrańska, Reiter & Posmyk (2016), Cui et al. (2017), and Li et al. (2017) reported a similar increase in the activities of antioxidant enzymes and reduce ROS in melatonin-treated pea (*Pisum sativum)*, watermelon, and wheat under abiotic stresses. Therefore, our results imply that melatonin treatment decreased the drought-mediated harmful effects on maize seedlings and increase its drought tolerance.

Furthermore, the production of different compatible solutes, commonly referred to as osmoprotectants is also an important strategy of plants to tolerate abiotic stresses (Dawood & El-Awadi, 2015; Zeng et al., 2018). The osmoprotectants including soluble proteins, proline, and total free amino acids play a prospective role to protect plants from stresses through contribution to cellular osmotic adjustment, stabilization of enzymes and proteins detoxification of ROS and protecting membrane integrity (Dorothea & Ramanjulu, 2005; Dawood & El-Awadi, 2015). Proline, as an osmotic protectant, contributes to the cells turgor maintenance under stress (Tiwari et al., 2010) and facilitates the synthesis of important proteins that otherwise are essential for stress responses (Iyer & Caplan, 1998; Dawood & El-Awadi, 2015). Melatonin applications substantially enhanced the proline and soluble protein concentration in maize seedlings under drought stress (Figs. 10 and 11), representing the potential efficacy of melatonin to effectively cope with the drought stress. Earlier studies reported that the application of an appropriate concentration of melatonin to *Citrus aurantium* L. seedlings and *Brassica napus* L. under abiotic stress significantly improved the concentrations of compatible solutes (Kostopoulou et al., 2015; Zeng et al., 2018). Remarkably, a higher concentration of melatonin when applied as foliar spray enhanced the gas exchange parameters improved the activities of antioxidant enzymes, soluble protein, and proline concentration but the higher concentration dampen the beneficial effects of melatonin when applied as a soil drench. Previous studies of melatonin pretreatment in rice (Liang et al., 2015) and brassica (Zeng et al., 2018) showed that low concertation of melatonin had the most beneficial effects, while higher concentrations had adverse effects. Nevertheless, a higher concentration of melatonin has been proposed to have a protective role in the photosynthetic apparatus against drought stress when applied along with irrigation water (Fleta-Soriano et al., 2017). Hence, based on the results of our present study, along with the observations of previous researchers, it is obvious that mitigation potential of melatonin is closely related to its appropriate concentration based on the application method.

### Conclusion

Overall, our results revealed that application of melatonin might be an efficient approach for improving tolerance of maize seedlings under drought stress. It primarily contributed to strengthening the antioxidant defense system of maize seedlings that resulted in reducing the drought-induced oxidative damages, as indicated by reduced MDA and H_2_O_2_ concentration. It is also enhanced the photosynthetic efficiency by protecting the pigments from degradation in maize seedlings under drought conditions. Application of melatonin also increases the concentration of soluble proteins and proline which work as osmoprotectants. Taken together, melatonin has a protective role in the maize seedlings grown under drought stress conditions and its optimum concentration will be helpful in elevating drought stress in many crops.

##  Supplemental Information

10.7717/peerj.7793/supp-1 Data S1 Raw data: each dataset consisted of 3 replicates

10.7717/peerj.7793/supp-2 Figure S1 Effects of melatonin on root phenotyping in maize seedlings under drought stress For Foliar application, FCK1 indicate well-watered, FCK2, drought stress; FM1, FM2, FM3, and FM4 indicates melatonin application at the rate of 25, 50, 75, 100 µM, respectively. For soil drench application, DCK1 indicates well-watered, DCK2, drought stress; DM1, DM2, DM3, and DM4 indicates melatonin application at the rate of 25, 50, 75, 100 µM.

## Additional Information and Declarations

### Data Availability

The following information was supplied regarding data availability: The raw data are available in the Supplemental File.
