# Supplementary figures and images for: Exogenous melatonin confers drought stress by promoting plant growth, photosynthetic capacity and antioxidant defense system of maize seedlings
Source: PeerJ. 2019 Oct 11;7:e7793. doi: 10.7717/peerj.7793 (PMC6791350; doi:10.7717/peerj.7793)

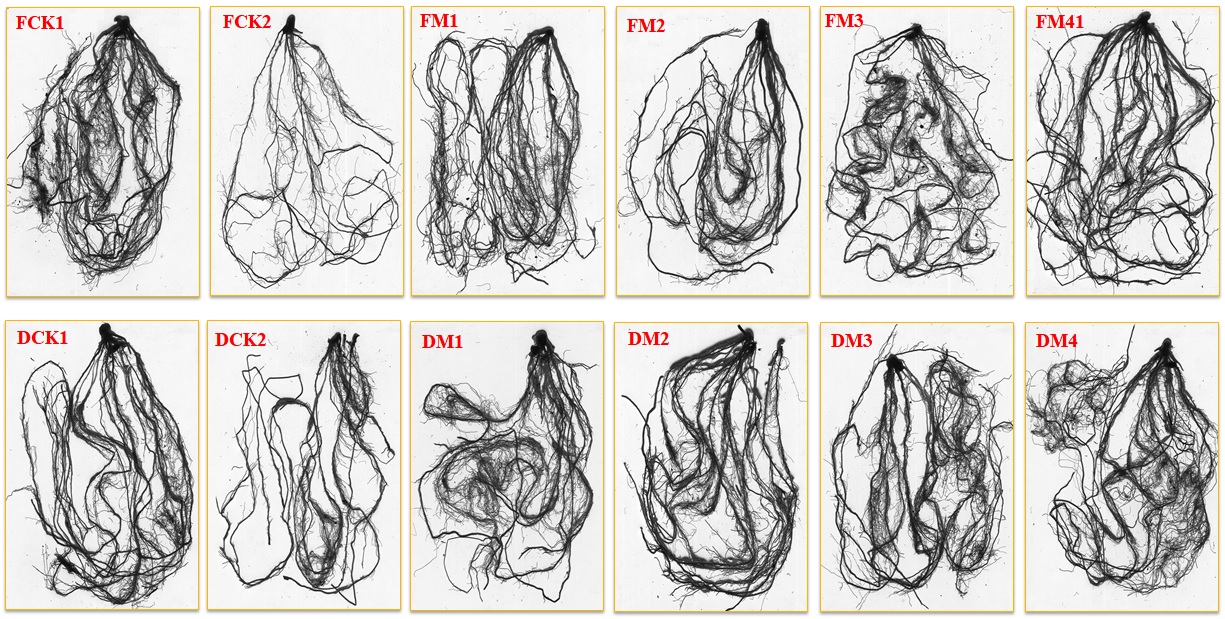

Supplement: Figure S1 — For Foliar application, FCK1 indicate well-watered, FCK2, drought stress; FM1, FM2, FM3, and FM4 indicates melatonin application at the rate of 25, 50, 75, 100 µM, respectively. For soil drench application, DCK1 indicates well-watered, DCK2, drought stress; DM1, DM2, DM3, and DM4 indicates melatonin application at the rate of 25, 50, 75, 100 µM. [file peerj-07-7793-s002.jpg]
